# Supplementary material for: Myeloid p38α signaling promotes intestinal IGF‐1 production and inflammation‐associated tumorigenesis
Source: EMBO Mol Med. 2018 Jun 15;10(7):e8403. doi: 10.15252/emmm.201708403 (PMC6034132; doi:10.15252/emmm.201708403)
Supplement: Supplementary file 1 — Appendix [file EMMM-10-e8403-s001.pdf]

Appendix

Myeloid p38α signaling promotes intestinal IGF-1 production and inflammation-associated tumorigenesis

Catrin Youssif, Monica Cubillos-Rojas, Mònica Comalada, Elisabeth Llonch, Cristian Perna, Nabil Djouder and Angel R. Nebreda

Table of contents

Appendix Figure S1. Downregulation of p38α in myeloid cells reduces inflammation in the tumor microenvironment.

Appendix Figure S2. Mice with p38α-deficient myeloid cells show reduced DSS induced colitis and decreased leukocyte recruitment during intestinal inflammation.

Appendix Figure S3. IGF-1 signaling promotes DSS-induced epithelial damage and inflammation.

Appendix Figure S4. Chemical inhibition of IGF-1 signaling ameliorates DSS-induced intestinal inflammation.

Appendix Figure S5. Effect of myeloid p38α on chemokine production in the colon and HSCs in the bone marrow.

Appendix Table S1. Primers used for PCR

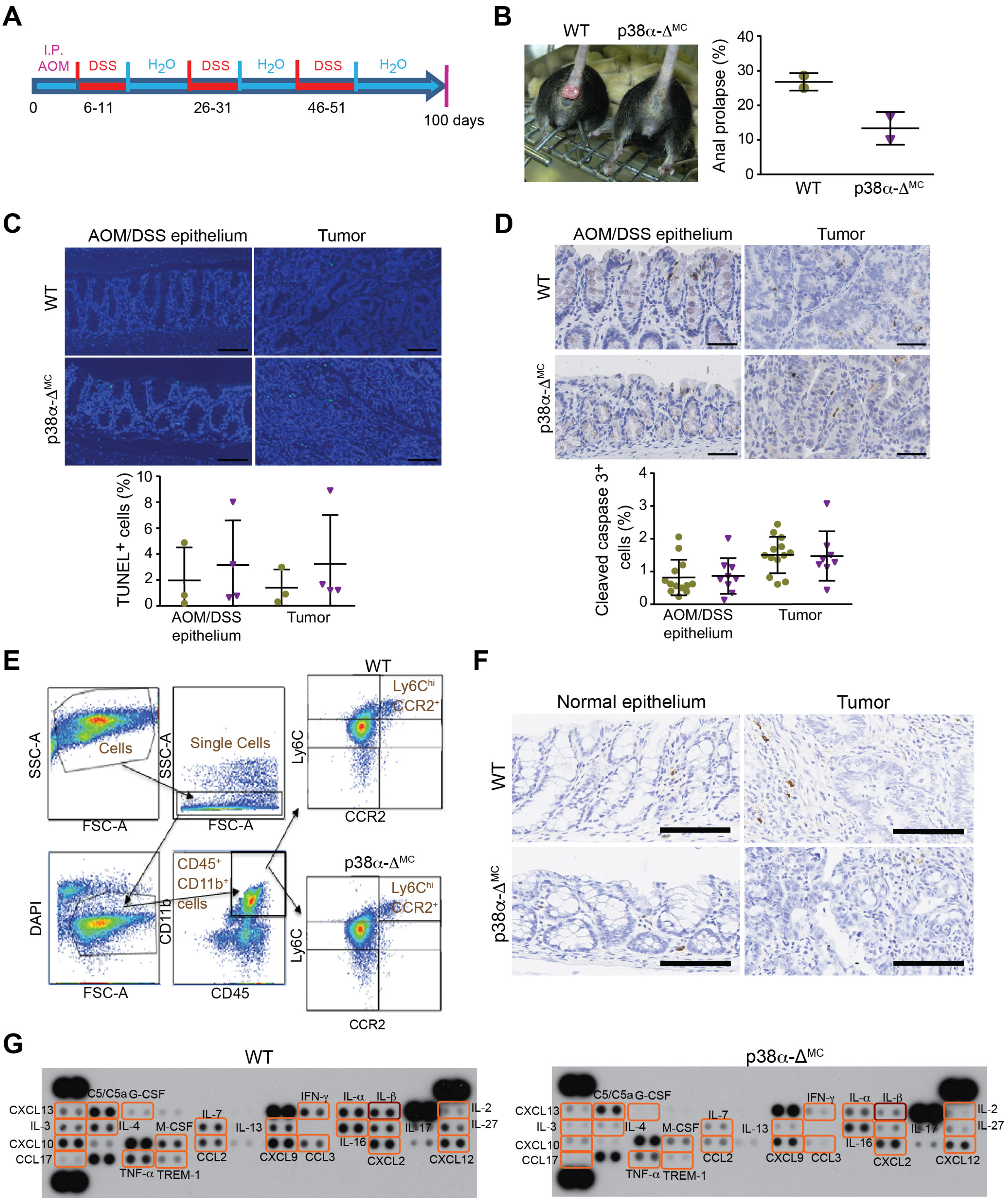

Appendix Figure S1. Downregulation of p38α in myeloid cells reduces inflammation in the tumor microenvironment.

A Schematic representation of the AOM/DSS protocol to induce colitis-associated colon cancer. Animals were sacrificed at day 100.

B Representative examples of anorectal prolapse and percentage of prolapse frequency of two experiments.

C Representative sections from AOM/DSS treated colon epithelia and colon tumors stained for TUNEL. Quantifications are shown in the histogram ($n \geq 3$). Scale bars, 100 μm.

D Representative sections from AOM/DSS treated colon epithelia and colon tumors stained for cleaved Caspase 3. Quantifications are shown in the histogram ($n \geq 8$). Scale bars, 100 μm.

E Representative FACS analysis for the selection of inflammatory monocytes in the bone marrow.

F Representative sections from normal colon epithelia and colon tumors stained for F4/80 that correspond to higher magnifications of the pictures in Figure 1E. Scale bars, 100 μm.

G A mouse cytokine antibody array was interrogated using pools of 3 mm tumors derived from AOM/DSS treated mice either WT or p38α-Δ^{MC} (n=8/genotype). Quantifications are shown in Figure 1F.

Statistical analysis was performed by using Mann-Whitney test for the comparison of two groups or ANOVA using Bonferroni post-hoc correction for multiple groups. Data are expressed as the average±SD.

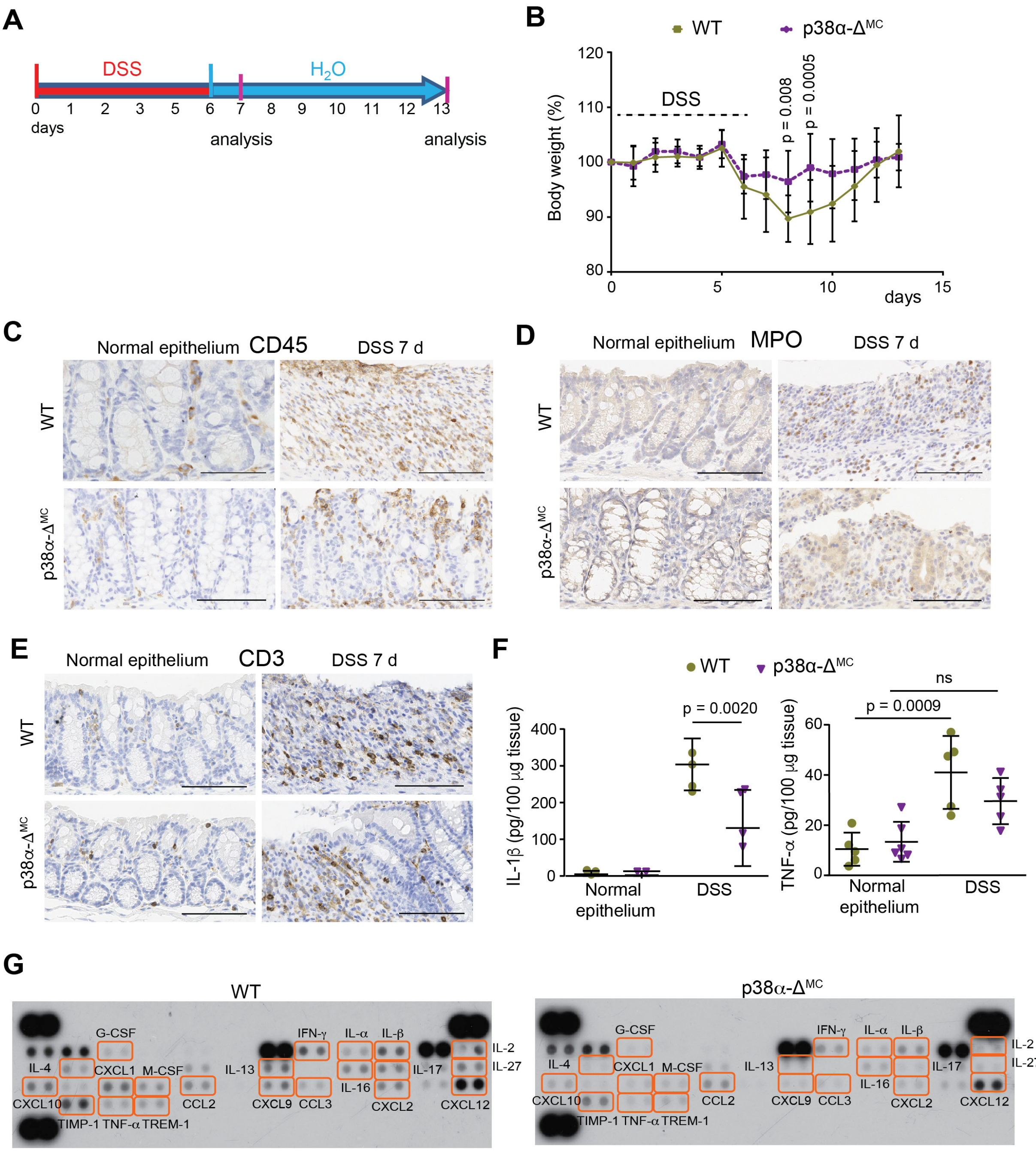

Appendix Figure S2. Mice with p38α-deficient myeloid cells show reduced DSS induced colitis and decreased leukocyte recruitment during intestinal inflammation.

A Schematic representation of the experimental design for DSS-induced acute colitis. Animals were sacrificed at days 7 and 13.

B Body weights in DSS-treated mice were recorded daily ($n \geq 9$).

C-E Representative colon sections stained for CD45 (C), MPO (D) and CD3 (E) from untreated mice or mice treated with DSS for 6 days and analyzed at day 7. Higher magnification of Figures EV2B-2D. Scale bars, 100 μm.

F Colon lysates from mice untreated or treated with DSS for 6 days were analyzed at day 7 by ELISA for IL-1β and TNF-α expression ($n \geq 5$).

G A mouse cytokine antibody array was interrogated using pools of whole colon extracts derived from DSS treated mice either WT or p38α-Δ^{MC} at day 7 (n=8/genotype). Quantifications are shown in Figure 2E.

Statistical analysis was performed by ANOVA using Bonferroni post-hoc correction for multiple groups. Data are expressed as the average±SD.

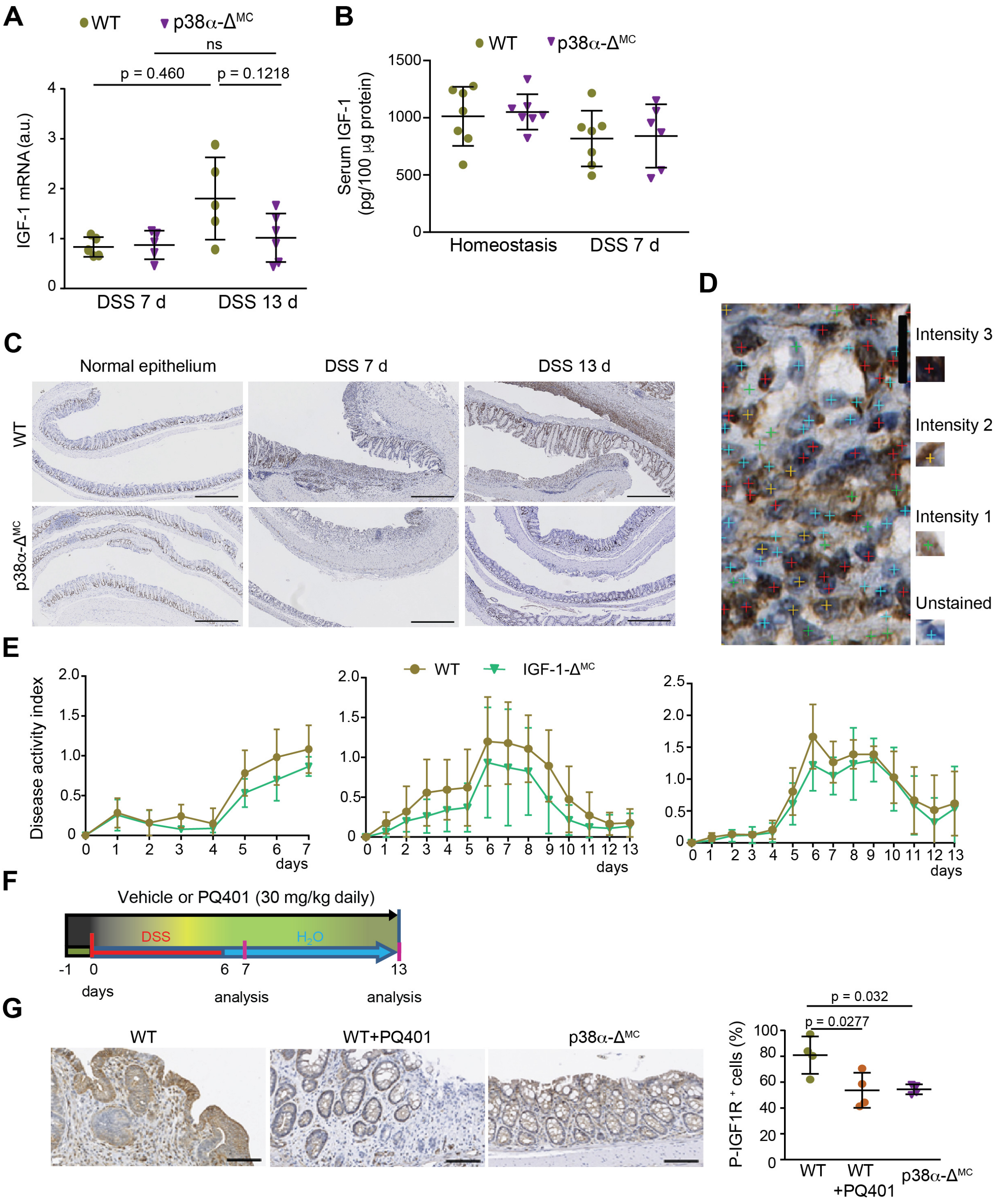

Appendix Figure S3. IGF-1 signaling promotes DSS-induced epithelial damage and inflammation.

A Colon lysates obtained from mice either untreated or treated with DSS for 6 days were analyzed at the indicated days to measure IGF-1 mRNA levels by qRT-PCR (n ≥ 4).

B Serum obtained from mice either untreated or treated with DSS for 6 days were analyzed at day 7 to measure IGF-1 protein levels by ELISA (n ≥ 6).

C Representative colon sections from mice either untreated or treated with DSS for 6 days were analyzed at the indicated times for Ki67 staining. Scale bars, 500 μm.

D Representative examples of cells stained for phospho-IGF1R and classified as unstained or stained with low (intensity 1), moderate (intensity 2) and high (intensity 3) intensities using the Tmarker software. Scale bar, 20 μm.

E Single experiments used for the pooled data shown in Figure EV3C. Disease activity index was recorded daily during DSS-induced colitis. Left panel $n \geq 3$, middle panel $n = 6$, right panel $n \geq 22$.

F Schematic representation of the protocol used to test the effect of the IGF1R inhibitor PQ401 in mice with DSS-induced acute colitis. Animals were sacrificed at day 7 and 13.

G Representative images of colon sections stained for phospho-IGF1R at day 13 after DSS and PQ401 treatment. Quantifications are shown in the histogram ($n = 4$). Scale bars, 100 μm.

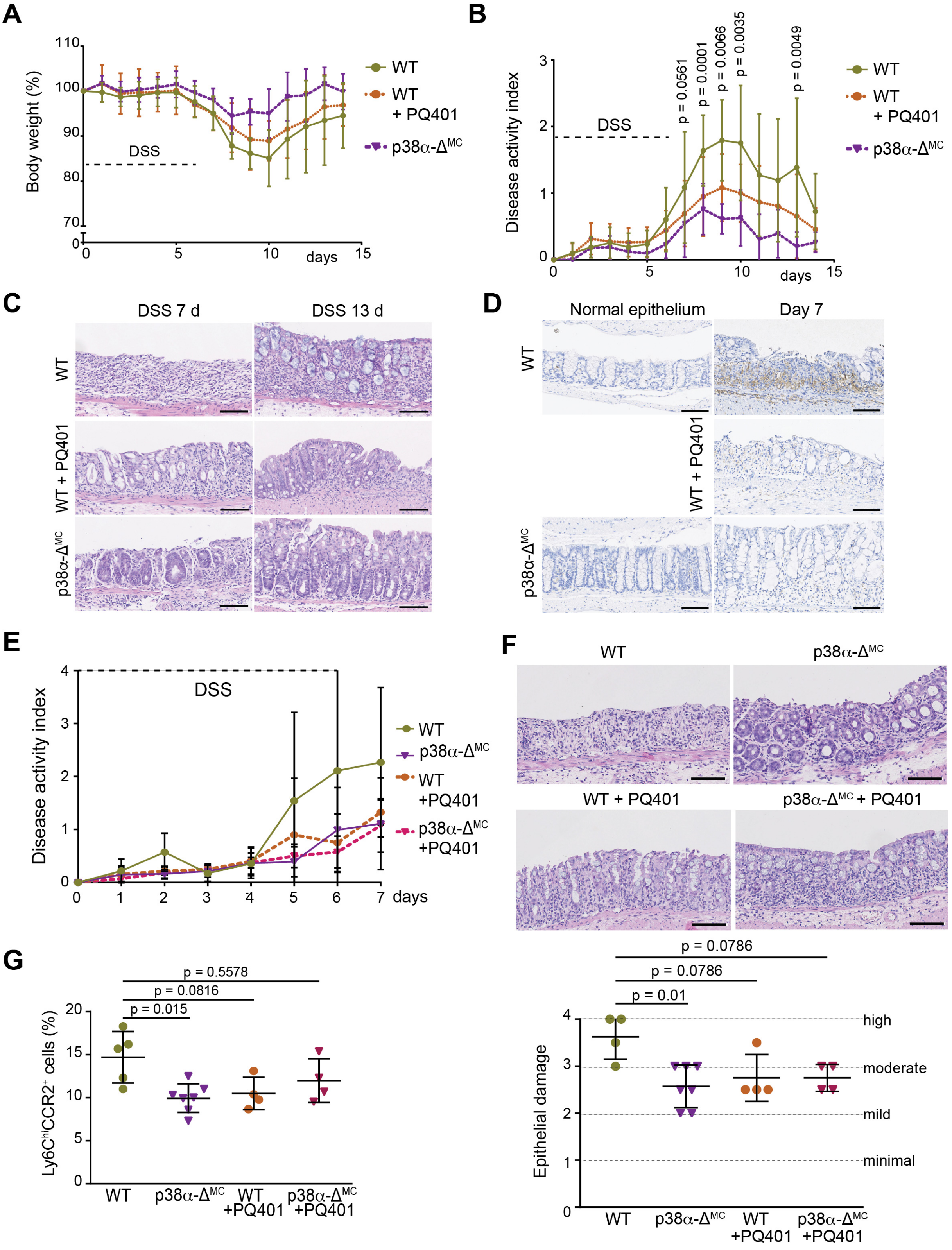

Appendix Figure S4. Chemical inhibition of IGF-1 signaling ameliorates DSS-induced intestinal inflammation.

A-B WT mice treated with the IGF1R inhibitor PQ401 or vehicle, and p38α-Δ^{MC} mice treated with vehicle were analyzed daily for body weight (**A**) and Disease activity index (**B**) during DSS-induced colitis (n ≥ 7).

C Representative H&E-stained colon sections from mice treated with either PQ401 or vehicle, and with DSS for 6 days were analyzed at the indicated days. Scale bars, 100 µm.

D Representative F4/80 staining of colon sections from untreated mice or mice treated with DSS and either PQ401 or vehicle. Scale bars, 100 µm.

E Disease activity index was recorded daily during DSS-induced colitis in mice treated with either PQ401 or vehicle ($n \geq 13$).

F Epithelial damage was evaluated in H&E-stained colon sections from mice treated with DSS and either PQ401 or vehicle, and analyzed at day 7. Quantifications are shown in the histogram ($n \geq 4$). Scale bars, 100 µm.

G Mice treated with DSS for 6 days and either PQ401 or vehicle, were analyzed at day 7 to determine the percentage of $Ly6C^{hi}CCR2^{+}$ cells within the bone marrow cells that were alive and $CD45^{+}CD11b^{+}$ ($n \geq 4$).

Statistical analysis was performed by ANOVA using Bonferroni post-hoc correction for multiple groups. Data are expressed as the average±SD.

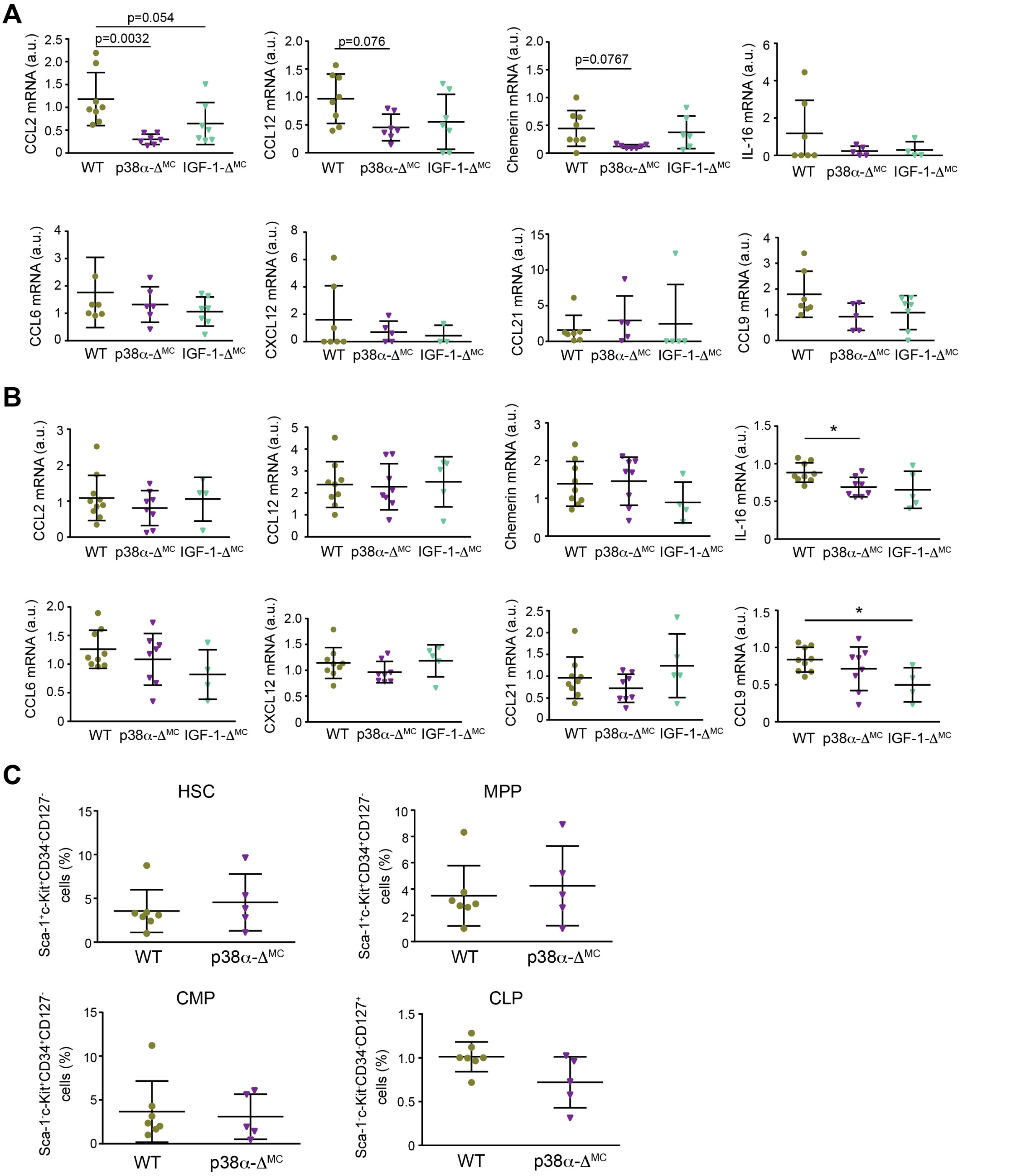

Appendix Figure S5. Effect of myeloid p38α on chemokine production in the colon and HSCs in the bone marrow.

A-B Relative levels for the indicated mRNAs in isolated intestinal macrophages (**A**) and whole colons (**B**) from WT, p38α-Δ^{MC} or IGF-1Δ^{MC} untreated mice were determined by qRT-PCR ($n \geq 4$).

C The bone marrows of untreated mice were analyzed by first removing differentiated blood cells with the so-called lineage cocktail, which contains antibodies against differentiation markers (Lin^{-} selection). The percentages of cells positive or negative for the indicated markers are indicated from the total Lin^{-} cell population ($n \geq 5$). Statistical analysis was performed by using Mann-Whitney test for the comparison of two groups or ANOVA using Bonferroni post-hoc correction for multiple groups. Data

are expressed as the average±SD. MPP, multipotent progenitor; CMP, common myeloid progenitor; CLP, common lymphoid progenitor

Appendix Table S1. Primers used for PCR

**Gene**	**Forward (5'-3')**	**Reverse (5'-3')**
CCL2	TTAAAAACCTGGATCGGAACCAA	GCATTAGCTTCAGATTTACGGGT
CCL21	GTGATGGAGGGGGTCAGGA	GGGATGGGACAGCCTAAACT
CCL6	TATCCTTGTGGCTGTCCTTGG	TTACATGGGATCTGTGTGGCA
CCL9	CCCTCTCCTTCCTCATTCTTACA	AGTCTTGAAAGCCCATGTGAAA
Chemerin	GCTGATCTCCCTAGCCCTATG	CCAATCACACCACTAACCACTTC
CXCL12	TGCATCAGTGACGGTAAACCA	TTCTTCAGCCGTGCAACAATC
GAPDH	CTTCACCACCATGGAGGAGGC	GGCATGGACTGTGGTCATGAG
IGF-1	CTGGACCAGAGACCCTTTGC	GGACGGGGACTTCTGAGTCTT
IL-16	AAGAGCCGGAAATCCACGAAA	GTCTCAAAAGGGTCAGGGTACT
CCL12	ATTTCCACACTTCTATGCCTCCT	ATCCAGTATGGTCCTGAAGATCA
Mapk14 (p38α) exon 12	GCCCTCCCTCACTTCAGGAG	TGTGCTCGGCACTGGAGACC
Mapk14 (p38α) exon 2	GCATCGTGTGGCAGTTAAGA	GTCCTTTTGGCGTGAATGAT
