# Supplementary material for: Myeloid p38α signaling promotes intestinal IGF‐1 production and inflammation‐associated tumorigenesis
Source: EMBO Mol Med. 2018 Jun 15;10(7):e8403. doi: 10.15252/emmm.201708403 (PMC6034132; doi:10.15252/emmm.201708403)
Supplement: Supplementary file 2 — Expanded View Figures PDF [file EMMM-10-e8403-s002.pdf]

Expanded View Figures

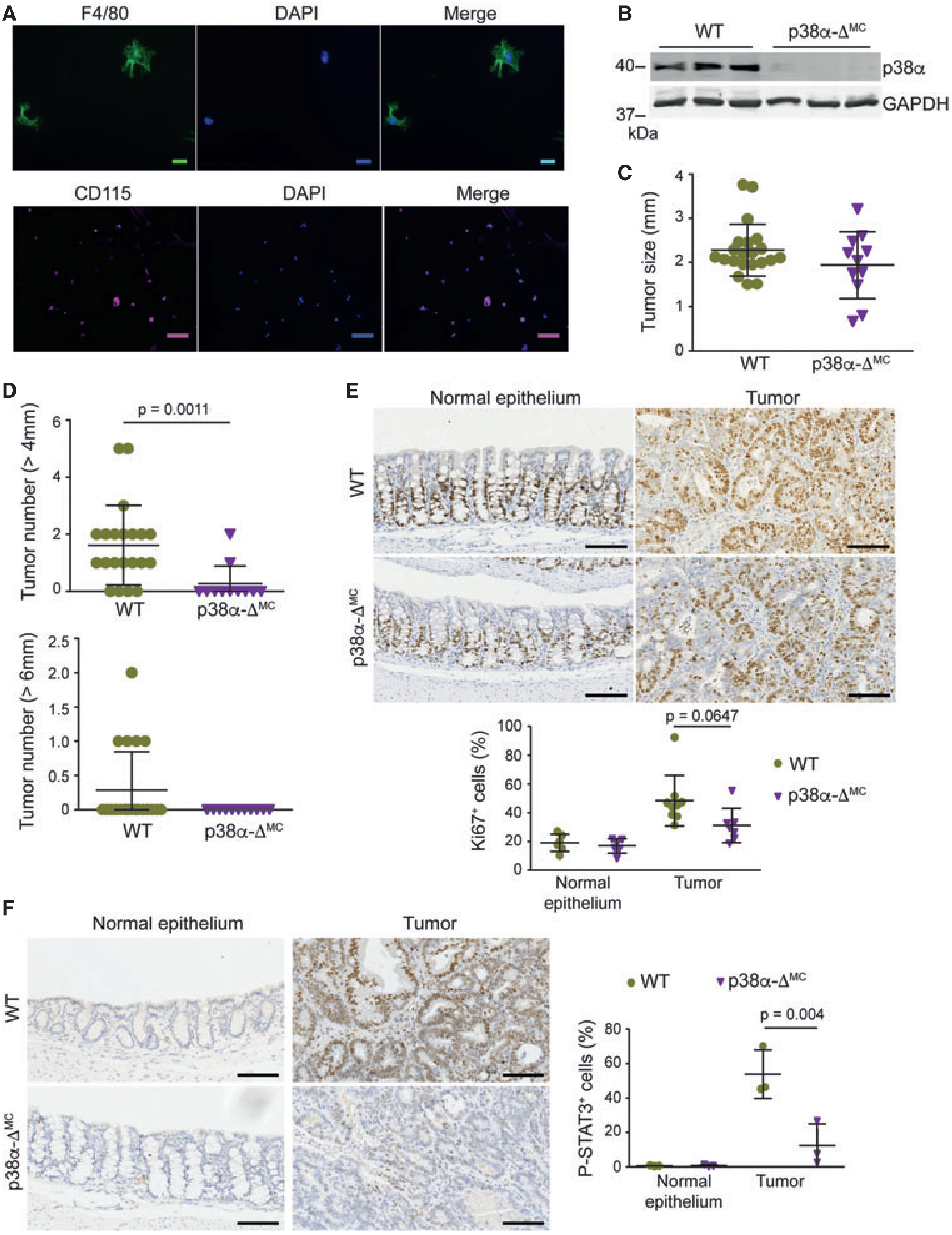

Figure EV1.

Figure EV1. Downregulation of p38α in myeloid cells reduces inflammation in the tumor microenvironment.

A Representative images of intestinal macrophages isolated from the colonic lamina propria and stained for F4/80 and CD115. Scale bars, 10 μm (F4/80) and 100 μm (CD115).
B Representative immunoblots of p38α in lysates from peritoneal macrophages.
C Average tumor size ($n \geq 11$).
D Number of tumors > 4 and >6 mm in AOM/DSS-treated mice ($n \geq 11$).
E, F Representative sections from normal colon epithelia and colon tumors stained for Ki67 ($n \geq 6$) (E) and phospho-STAT3 ($n \geq 3$) (F). Quantifications are shown in the histogram. Scale bars, 100 μm.

Data information: Statistical analysis was performed by using Mann–Whitney test for the comparison of two groups or ANOVA using Bonferroni *post hoc* correction for multiple groups. Data are expressed as the average ± SD.

Figure EV2. Mice with p38α-deficient myeloid cells show reduced DSS-induced colitis and decreased leukocyte recruitment during intestinal inflammation.

A Representative images of H&E-stained colon sections from animals either untreated or treated with DSS for 6 days and analyzed at the indicated days. Scale bars, 100 μm.
B–D Representative colon sections stained for CD45 (B), MPO (C), and CD3 (D) from untreated mice or mice treated with DSS for 6 days and analyzed at day 7 ($n \geq 5$). Quantifications are shown in the histogram. Scale bars, 100 μm.

Data information: Statistical analysis was performed by ANOVA using Bonferroni *post hoc* correction. Data are expressed as the average ± SD.

© 2018 The Authors

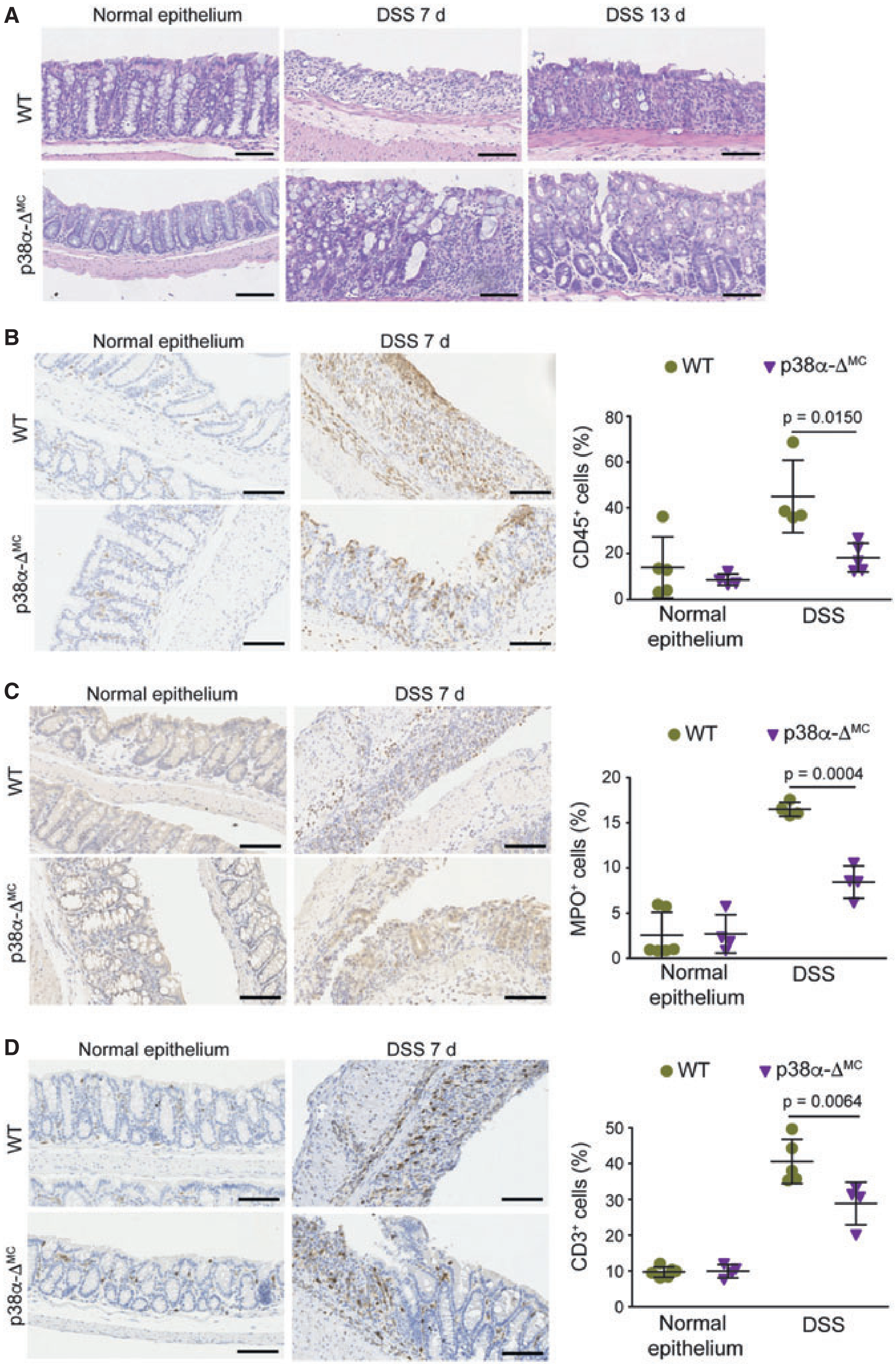
A
Normal epithelium
DSS 7 d
DSS 13 d
WT
p38α-Δ^MC
B
Normal epithelium
DSS 7 d
WT
p38α-Δ^MC
WT
p38α-Δ^MC
CD45⁺ cells (%)
p = 0.0150
80
60
40
20
0
Normal epithelium
DSS
C
Normal epithelium
DSS 7 d
WT
p38α-Δ^MC
WT
p38α-Δ^MC
MPO⁺ cells (%)
p = 0.0004
20
15
10
5
0
Normal epithelium
DSS
D
Normal epithelium
DSS 7 d
WT
p38α-Δ^MC
WT
p38α-Δ^MC
CD3⁺ cells (%)
p = 0.0064
50
40
30
20
10
0
Normal epithelium
DSS

Figure EV2.

Figure EV3. Downregulation of IGF-1 in myeloid cells reduces susceptibility to intestinal inflammation.

A Relative IGF-1 mRNA levels in peritoneal macrophages ($n \geq 8$).

B, C Body weight (B) and disease activity index (C) were recorded daily during DSS-induced colitis ($n \geq 31$). This is a pool from three experiments, which are shown individually in Appendix Fig S3C.

D Representative images of H&E-stained sections from mice either untreated or treated with DSS for 6 days and analyzed at the indicated days. Scale bars, 100 µm

E, F Representative colon sections stained for phospho-STAT3 (E) or phospho-IGF1R (F) from mice treated with DSS for 6 days and analyzed at the indicated days. Quantifications are shown in the histograms ($n \geq 7$). Scale bars, 100 µm.

Data information: Statistical analysis was performed by using Mann–Whitney test for the comparison of two groups or ANOVA using Bonferroni *post hoc* correction for multiple groups. Data are expressed as the average ± SD.

 © 2018 The Authors

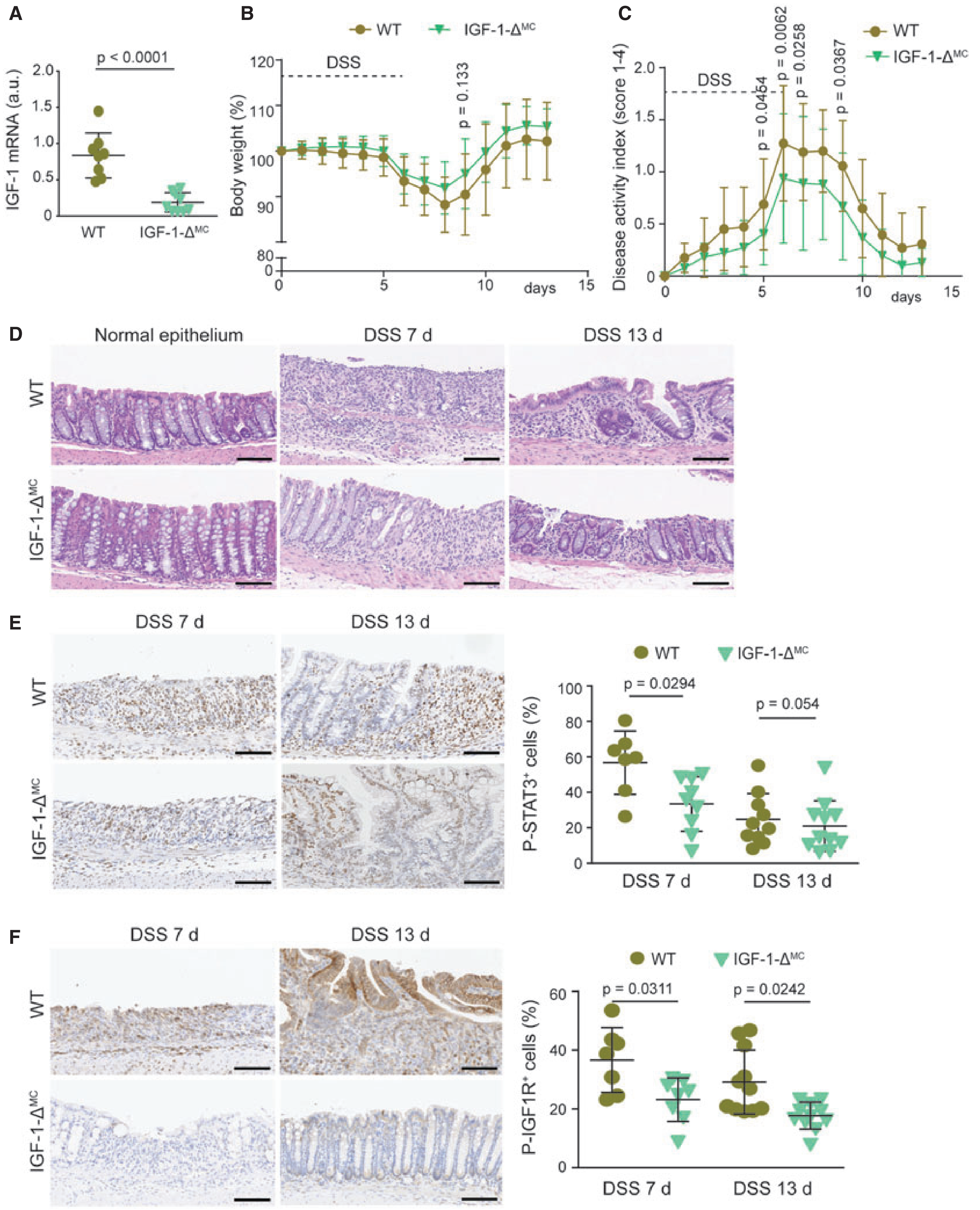
A
p < 0.0001
IGF-1 mRNA (a.u.)
2.0
1.5
1.0
0.5
0
WT
IGF-1-Δ^MC
B
WT
IGF-1-Δ^MC
DSS
p = 0.133
Body weight (%)
120
110
100
90
80
0
0
5
10
days
15
C
WT
IGF-1-Δ^MC
DSS
p = 0.0454
p = 0.0062
p = 0.0258
p = 0.0367
Disease activity index (score 1-4)
2.0
1.5
1.0
0.5
0
0
5
10
days
15
D
Normal epithelium
DSS 7 d
DSS 13 d
WT
IGF-1-Δ^MC
E
DSS 7 d
DSS 13 d
WT
IGF-1-Δ^MC
WT
IGF-1-Δ^MC
p = 0.0294
p = 0.054
P-STAT3+ cells (%)
100
80
60
40
20
0
DSS 7 d
DSS 13 d
F
DSS 7 d
DSS 13 d
WT
IGF-1-Δ^MC
WT
IGF-1-Δ^MC
p = 0.0311
p = 0.0242
P-IGF1R+ cells (%)
60
40
20
0
DSS 7 d
DSS 13 d

Figure EV3.

Figure EV4. Myeloid p38α downregulation reduces chemokine expression and inflammatory cell recruitment from the bone marrow.

A Percentage of $Ly6C^{hi}$ and $CCR2^+$ in bone marrow cells that were alive and $CD45^+$ $CD11b^+$ from untreated WT and p38α-Δ^{MC} mice (n = 11).
B Percentage of $Ly6C^{hi}$ and $CCR2^+$ in bone marrow cells that were alive and $CD45^+$ $CD11b^+$ from untreated WT and IGF-1-Δ^{MC} mice ($n \geq 5$).
C A mouse chemokine antibody array was interrogated using pools of whole colon extracts derived from non-stimulated mice either WT or p38α-Δ^{MC} (n = 5/genotype). Quantifications are shown in the lower panel. Arbitrary units (a.u.) are referred to the expression level of each chemokine in WT mice, which was given the value of 1.
D Percentage of $CD45^+$ $CD11b^+$ cells in the live cell population from colons of untreated WT and p38α-Δ^{MC} mice (n = 8).
E The indicated cell populations were sorted from the bone marrow of mice, and genomic DNA was analyzed by qPCR for the levels of floxed exon 2 versus exon 12 (as a control) of the *Mapk14* gene encoding p38α ($n \geq 5$).
F Representative bone marrow sections stained for phospho-IGF1R from untreated mice. Scale bars, 100 μm.
G Bone marrow sections from untreated mice ($n \geq 9$) were stained for phospho-IGF1R. The histograms show the quantification of all cells that were stained for phospho-$IGF1R^+$ (left panel) or only cells that show moderate (2^+) and high (3^+) intensities (right panel).

Data information: Statistical analysis was performed by using Mann–Whitney test for the comparison of two groups or ANOVA using Bonferroni *post hoc* correction for multiple groups. Data are expressed as the average ± SD.

 © 2018 The Authors

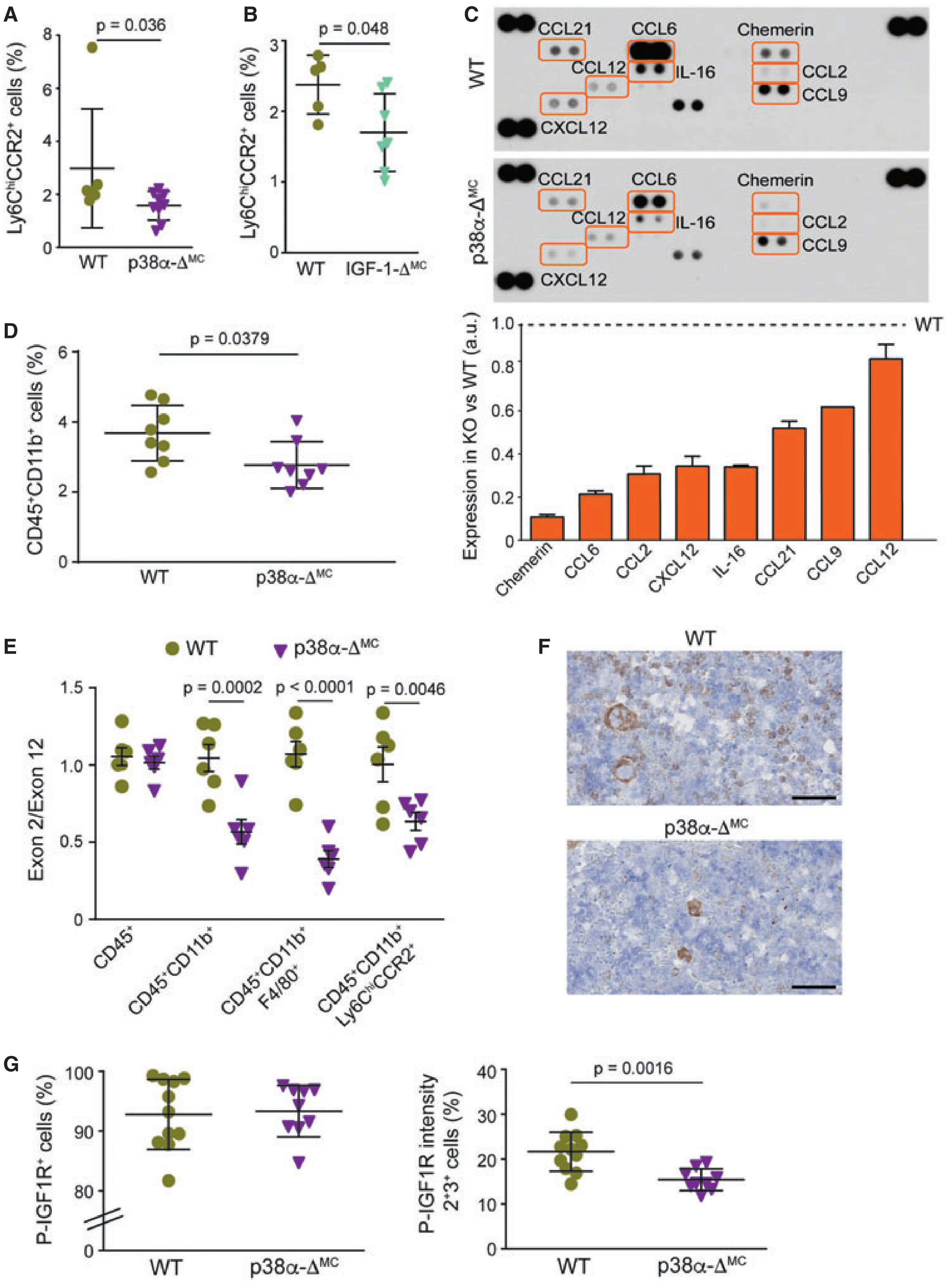
A
p = 0.036
Ly6C^hiCCR2^+ cells (%)
WT
p38α-Δ^MC
B
p = 0.048
Ly6C^hiCCR2^+ cells (%)
WT
IGF-1-Δ^MC
C
WT
CCL21
CCL6
Chemerin
CCL12
IL-16
CCL2
CCL9
CXCL12
p38α-Δ^MC
Expression in KO vs WT (a.u.)
Chemerin
CCL6
CCL2
CXCL12
IL-16
CCL21
CCL9
CCL12
D
p = 0.0379
CD45^+CD11b^+ cells (%)
WT
p38α-Δ^MC
E
WT
p38α-Δ^MC
p = 0.0002
p < 0.0001
p = 0.0046
Exon 2/Exon 12
CD45^+
CD45^+CD11b^+
CD45^+CD11b^+ F4/80^+
CD45^+CD11b^+ Ly6C^hiCCR2^+
F
WT
p38α-Δ^MC
G
P-IGF1R^+ cells (%)
WT
p38α-Δ^MC
p = 0.0016
P-IGF1R intensity 2^+3^+ cells (%)
WT
p38α-Δ^MC

Figure EV4.

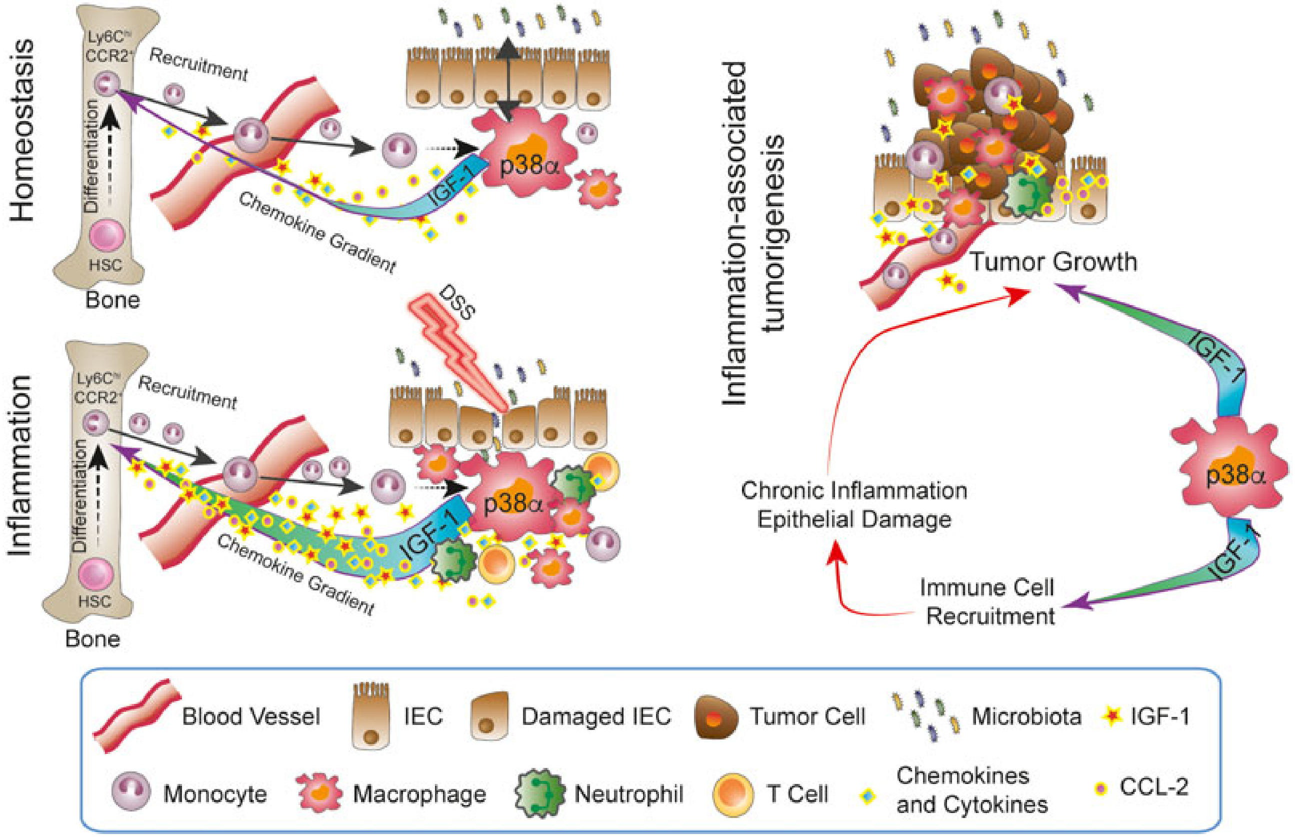

Figure EV5. The myeloid p38α-IGF-1 axis in the intestine under healthy and pathological conditions.

Commensal microbiota lead to a situation of tightly controlled inflammation, which involves constant recruitment of monocytes from hematopoietic stem cells (HSCs) in the bone marrow to the intestine. Intestinal macrophages rely on p38α to control the expression of IGF-1 and chemokines that are important for this process. Under pathological conditions that increase intestinal inflammation, immune cell recruitment is boosted and a vicious cycle may start that leads to the development of chronic inflammation and eventually, in certain cases, to the formation of tumors. IGF-1 produced by myeloid cells may also facilitate tumorigenesis by acting directly on tumor cells.

© 2018 The Authors
